# Human microRNA target analysis and gene ontology clustering by GOmir, a novel stand-alone application

**DOI:** 10.1186/1471-2105-10-S6-S20

**Published:** 2009-06-16

**Authors:** Maria G Roubelakis, Pantelis Zotos, Georgios Papachristoudis, Ioannis Michalopoulos, Kalliopi I Pappa, Nicholas P Anagnou, Sophia Kossida

**Affiliations:** 1 Cell & Gene Therapy Laboratory, Biomedical Research Foundation of the Academy of Athens, Soranou Efesiou 4, 11527, Athens, Greece; 2 Laboratory of Biology, University of Athens School of Medicine, Athens, Greece; 3 Bioinformatics & Medical Informatics Team, Biomedical Research Foundation of the Academy of Athens, Soranou Efesiou 4, 11527, Athens, Greece; 4 Ecole Polytechnique Fédérale de Lausanne, Switzerland; 5 MIT Computer Science and Artificial Intelligence Laboratory, The Stata Center, Building 32, 32 Vassar Street, Cambridge, MA 02139, USA; 6 First Department of Obstetrics and Gynecology, University of Athens School of Medicine, Athens, Greece

## Abstract

**Background:**

microRNAs (miRNAs) are single-stranded RNA molecules of about 20–23 nucleotides length found in a wide variety of organisms. miRNAs regulate gene expression, by interacting with target mRNAs at specific sites in order to induce cleavage of the message or inhibit translation. Predicting or verifying mRNA targets of specific miRNAs is a difficult process of great importance.

**Results:**

GOmir is a novel stand-alone application consisting of two separate tools: JTarget and TAGGO. JTarget integrates miRNA target prediction and functional analysis by combining the predicted target genes from TargetScan, miRanda, RNAhybrid and PicTar computational tools as well as the experimentally supported targets from TarBase and also providing a full gene description and functional analysis for each target gene. On the other hand, TAGGO application is designed to automatically group gene ontology annotations, taking advantage of the Gene Ontology (GO), in order to extract the main attributes of sets of proteins. GOmir represents a new tool incorporating two separate Java applications integrated into one stand-alone Java application.

**Conclusion:**

GOmir (by using up to five different databases) introduces miRNA predicted targets accompanied by (a) full gene description, (b) functional analysis and (c) detailed gene ontology clustering. Additionally, a reverse search initiated by a potential target can also be conducted. GOmir can freely be downloaded BRFAA.

## Background

microRNAS (miRNAs) are 20- to 23- nucleotide long single stranded RNAs that post-transcriptionally regulate gene expression [1,2]. miRNAs act as translation inhibitors of mRNA into protein and promote mRNA degradation. In this way, miRNAs play important role in various cell processes such as proliferation, differentiation, apoptosis, development, cancer and various other diseases [1,2] and thus represent potential targets for therapeutic applications. The biogenesis of miRNAs is a complicated process involving two different cellular compartments [3]. First, in the nucleus, a primary miRNA (pri-miRNA) is transcribed from the genomic DNA by RNA polymerase II. The size of this primary product varies from 100- to 1000- nucleotides in length. Then, the pri-miRNA is truncated by Drosha and DGCR8 to form a hairpin loop precursor called pre-miRNA [3]. The 60–70 nucleotide long pre-miRNA is loaded to Exportin 8 and Ran-GTP in order to be exported into the cytoplasm. A mature miRNA (20–23 nucleotides) is then released by the RNAse III endonuclease complex including Dicer and trans-activator RNA (tar)-binding protein TRBP [3]. The mature miRNA then inhibits translation of a miRNA into a protein by imperfect base pairing to one or more mRNA sequences [1,4,5]. The identification of human miRNAs and their respective targets is of great importance and involves both computational and experimental approaches [5]. Prediction servers such as TargetScan [6], miRanda [7], RNAhybrid [8], PicTar [9] and the recent one DIANA-MicroT 3.0 [10] give information for the miRNA-target interactions. Recent reports have described correlated computational expression of miRNA and their target mRNAs and proteins giving a detailed functional description of the latest [4,11]. Herein, we describe GOmir [12], a new stand-alone application for human miRNAs target prediction and ontology clustering, consisting of two different components, JTarget and TAGGO. JTarget combines the data from four different prediction databases (TargetScan, miRanda, RNAhybrid and PicTar) and also from the experimental database TarBase [13], whereas TAGGO gives detailed assignments from Gene Ontology (GO) resources to gene products. TAGGO uses one of the most reliable biological ontologies, the Gene Ontology, the main goal of which is to provide a well structured, precisely defined and controlled vocabulary for describing the roles of genes and gene products in any organism. GO was initiated back in 1998, as a collaborative effort to build consistency of gene product descriptions among different databases, initially including three model organisms. Since then, many plant, animal and microbial genomes have been assimilated [14]. GO was developed into three structured controlled vocabularies (ontologies) that describe gene products in terms of their associated biological processes, cellular components and molecular functions in a species-independent manner [14]. Thus, GOmir serves as a reliable tool for miRNA target prediction and more interestingly provides assignments from GO resources for these gene products, exploring in this way the functional aspects of miRNAs in more detail.

## Results

### User interface

**Figure 1:**
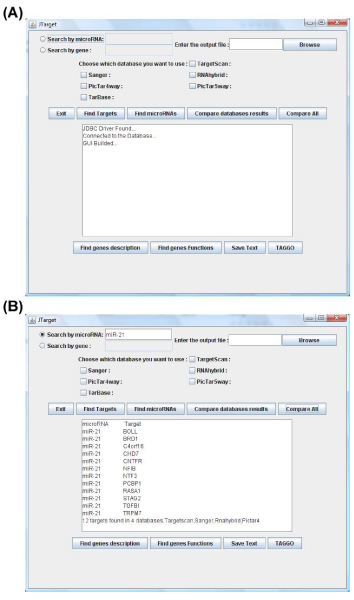
**JTarget main menu**. (A) Jtarget interface (B) The target genes of miR-21 as listed by the procedure "Compare All'' (4 databases).

JTarget main goal is to find the target genes of a given miRNA derived from 1 to 6 databases and compare any possible combination of the results from these databases. The available databases are TargetScan, miRanda, RNAhybrid, PicTar-4 way, PicTar-5 way and TarBase. Following the insertion of a specific miRNA name, 3 possible scenarios are given. The user can obtain the resulting target genes from: a) one database only, b) two or more databases or c) all combined databases. JTarget contains text fields, buttons and one central text area for the interaction with the user (Figure 1A). To find the target genes of a miRNA from a single database, the "Search by miRNA" option has to be selected and a miRNA name (e.g. miR-21) should be given. Then, a database (e.g. TargetScan) should be marked and the results are presented in the central text area by clicking the "Find Targets" button. The common results from 2 or more databases can be obtained by marking the desirable databases and clicking the "Compare database results" button. The available databases are: TargetScan, miRanda, RNAhybrid, PicTar-4 way, PicTar-5 way and the experimental one, TarBase. In this study, for the first time, the RNAhybrid database sets were introduced in a computational program.

To obtain the common target genes results for a given miRNA from all the databases (TargetScan, miRanda, RNAhybrid, and PicTar-4 way), only the "Compare All" button should be clicked without the necessity to select any databases (Figure 1B). The databases, that give no results for a given miRNA, are excluded from the procedure and the common target genes from the remaining databases are provided. The obtained results can be further compared to the experimentally supported targets provided, for a certain miRNA, by clicking the TarBase button. In addition, it is possible to perform a reverse search. The user may enter a gene symbol (e.g. *STAG2 *for STROMAL ANTIGEN 2) and obtain the common miRNAs of the selected databases for the given gene. The JTarget includes datasets from PicTar-4 way and the -5 way databases. Both databases contain target prediction for all human miRNAs based on conservation in mammals. The PicTar-4 way includes human, mouse, rat, dog species datasets, whereas the 5-way includes also chicken species datasets. However, the insertion of PicTar-5 way reduces the common target genes results to a significant degree. Therefore, we decided to exclude this database from the calculation of the common target genes at the "Compare All" procedure. The number of the predicted targets by searching one or more databases for miR-21, miR-31, miR-221 and miR-222 is shown in Table 1.

**Table 1:** Number of common targets found for several miRNAs from 1, 2, 4 OR 5 databases

miRNAs	TargetScan	TargetScan and miRanda	Compare All (4)	Five databases
miR-21	186	44	12	6
miR-31	192	20	7	0
miR-221	251	49	11	5
miR-222	251	40	10	7

The results can be saved either before the search is accomplished, by filling the "Enter the output file" text field, or by choosing "Save Text" after the completion of the search. Then, the descriptions of the resulted target genes can be found by the "Find genes description" application, where the full description of each gene is presented in the text area. In addition, the functions of each target gene can be found when the "Find genes functions" is clicked. The information about the genes description and functions is obtained by the DAVID Bionformatics database [15]. In Table 2, the descriptions of the common genes from 4 databases predicted for the miR-21 are described. The functions of the common target genes predicted by the JTarget "Find genes functions" procedure for miR-21 from all 5 databases are shown in Table 3. The blank cells next to certain genes mean that these genes are not contained in DAVID's gene function database.

**Table 2:** Description of the common target genes of miR-21 from 4 databases

Gene	Description
BOLL	BOL, BOULE-LIKE (DROSOPHILA)
BRD1	BROMODOMAIN CONTAINING 1
C4orf16	CHROMOSOME 4 OPEN READING FRAME 16
CHD7	CHROMODOMAIN HELICASE DNA BINDING PROTEIN 7
CNTFR	CILIARY NEUROTROPHIC FACTOR RECEPTOR
NFIB	NUCLEAR FACTOR I/B
NTF3	NEUROTROPHIN 3
PCBP1	POLY(RC) BINDING PROTEIN 1
RASA1	RAS P21 PROTEIN ACTIVATOR (GTPASE ACTIVATING PROTEIN RAS P21)
STAG2	STROMAL ANTIGEN 2
TGFBI	TRANSFORMING GROWTH FACTOR, BETA-INDUCED, 68KDA
TRPM7	TRANSIENT RECEPTOR POTENTIAL CATION CHANNEL, SUBFAMILY M, MEMBER 7

**Table 3:** Functions of the common target genes of mir_21 from 5 databases

Gene	Function
NFIB	NFIB is capable of activating transcription and replication.
C4orf16	
RASA1	RASA1 is an inhibitory regulator of the Ras-cyclic AMP pathway.
STAG2	STAG2 is a component of cohesin complex, required for the cohesion of sister chromatids after DNA replication.
NTF3	NTF3 promotes the survival of visceral and proprioceptive sensory neurons.
CHD7	

### TarBase comparative analyses

**Figure 2:**
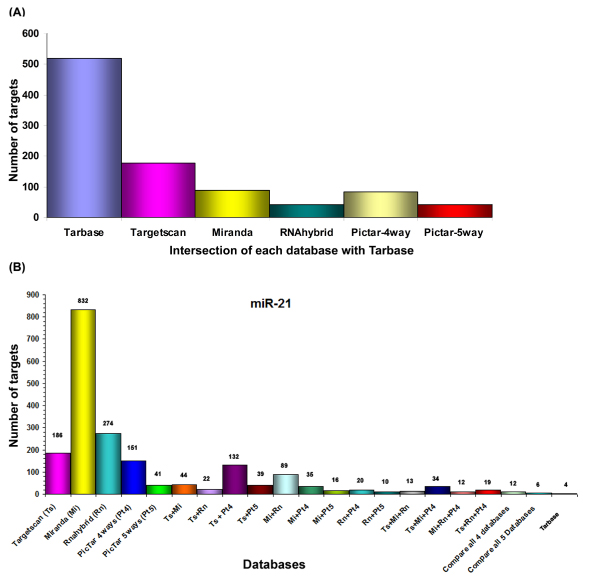
**Computational prediction validation by TarBase**. (A) Intersection of each prediction database with TarBase. The total number of common targets between each prediction database and TarBase for all the miRNAs available in TarBase was calculated. (B) The number of predicted targets for miR-21 by each predicting database or combinations of them. Experimentally supported targets for miR-21 are provided by TarBase.

As described in the previous section, TarBase was also inserted in the JTarget comparative module of GOmir. TarBase describes in full detail the experimentally supported targets of 111 miRNAs and also provides information for the nature of the experiments that were conducted to validate these results [13]. In order to understand the features of miRNA targeting and to validate experimentally the bioinformatics prediction, we performed comparative analyses using prediction databases (TargetScan, miRanda, RNAhybrid, PicTar-4 way and PicTar-5 way) and the experimental one, TarBase. An intersection analysis between each prediction database and TarBase was performed by calculating the total number of common targets between each prediction database and TarBase for all the miRNAs available in TarBase (Figure 2A). TargetScan predictions, as documented in Figure 2A, are validated experimentally in a higher rate compared to miRanda, RNAhybrid, PicTar-4 way and PicTar-5 way.

Using as an example the miR-21, TargetScan predicts 186 targets, whereas miRanda, RNAhybrid, PicTar-4 way and PicTar-5 way predict 832, 274, 151 and 41 targets, respectively for the same microRNA (Figure 2B). GOmir conducting analysis by comparing 4 databases (TargetScan, miRanda, RNAhybrid and PicTar-4 way) revealed 12 common predicted targets. However, TarBase has described up to date 4 experimentally supported targets (*PDCD4*, *TPM1*, *SERPINB5 *and *PTEN*) for miR-21, whereas *PDCD4 *was also predicted by GOmir comparative analysis of 3 out of 4 databases (TargetScan, miRanda and PicTar-4 way) and this may in turn explain the importance of selecting common targets between different predicting miRNA databases.

### Gene ontology analysis by TAGGO

**Figure 3:**
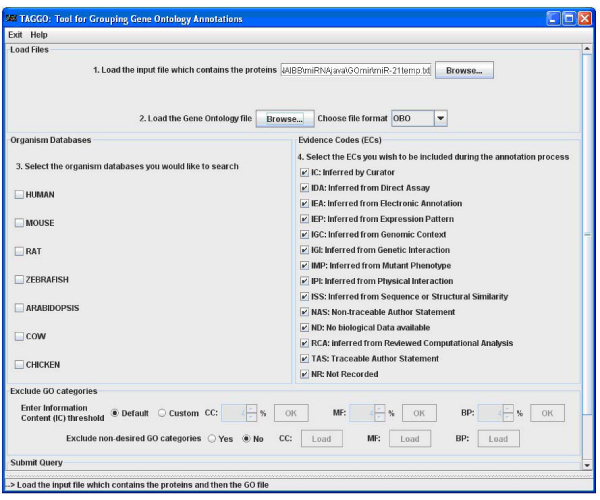
**TAGGO main menu**. TAGGO interface is presented.

**Figure 4:**
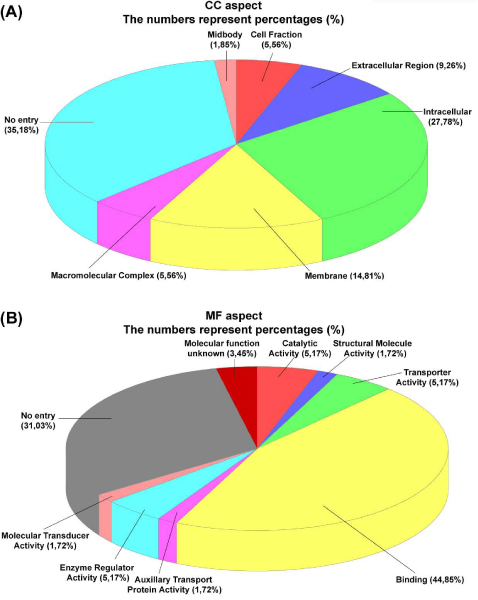
**TAGGO sample analysis. **(A) Subcellular destribution of the common target genes for miR-21 as derived from TargetScan and miRanda datasets. (B) Functional classification of the common target genes for miR-21 as derived from TargetScan and miRanda datasets.

Once a search is completed, a gene ontology clustering may be performed by clicking the "TAGGO" button. A temporary file is created from the output file of a JTarget search and is then used as an input file for TAGGO search. TAGGO interface consists of 5 steps (Figure 3). First, the user has to provide the path of the input file containing the proteins. When TAGGO is initiated through JTarget, the first step of TAGGO is done automatically, as the input file is provided directly by JTarget. The second step consists of the selection of the gene ontology file and its format. At the third step, the user is asked to choose the organism for which the clustering is going to be performed. However, for the present analysis the HUMAN checkbox should be selected. The user may then select the Evidence Codes to be included during the annotation process (all of them are included by default), may exclude some non-desired terms or may set a normalised information content threshold for the three aspects. The fifth step is to provide the program with an output directory. The output of the whole process is a directory called "Results", which includes the charts for the visualization of the output, three Venn lists for each GO aspect and some text files with information about the process and its results. For example, according to TAGGO results for the common target genes for miR-21 from TargetScan and miRanda, the most abundant GO term for Cellular Component GO aspect is GO:0005562 which corresponds to intracellular (Figure 4A) and for Molecular Function GO aspect is GO:0005488 which corresponds to binding functions, respectively (Figure 4B). A reverse search is possible to be performed in JTarget, using the "Search by gene" option, in order to find the complementary miRNAs for the given gene. Searches for common miRNAs from 2 to 5 databases are available as well.

### miR-21 regulates *TGF-β *target in Hela cells

**Figure 5:**
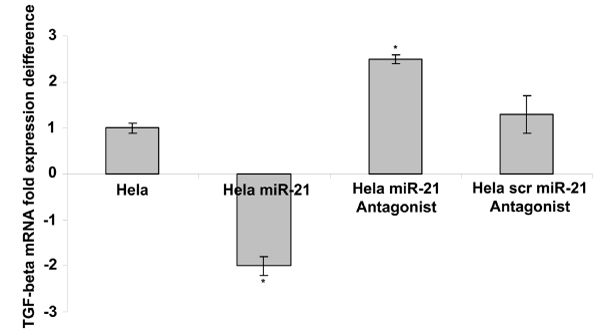
**TGF-*β *target regulation by miR-21 in Hela cells**. Semi-quantitative RT-PCR analysis for TGF-*β *in Hela cells transiently transfected with miR-21, miR-21 antagonist or miR-21 scambled antagonist. Values are shown as mean ± SEM for 3 independent experiments. Statistical analysis was performed using the Student's t-test (p < 0.05 and 0.04 for Hela transfected with mir-21 and Hela miR-21 antagonist, respectively)

A preliminary experimental approach was performed, in order to test whether a miRNA could regulate a predicted target by GOmir comparative analysis using 4 databases (TargetScan, miRanda, RNAhybrid and PicTar-4 way). *TGF-β *(transforming growth factor-beta) was predicted by GOmir as a target of miR-21 by 4 databases comparative analysis (Table 2). Hela cancer cell line is expressing *TGF-β *in a high level, thus was used as a model of *TGF-β *regulation by miR-21. In order to determine whether miR-21 functions as a possible regulator of *TGF-β*, Hela cells were transiently transfected with miR-21 mimic, miR-21 antagonist or miR-21 scrambled antagonist. The levels of miR-21 in transfected and non-transfected Hela cells were determined by real-time PCR and the transfection efficiency was established to 47–50% (data not shown). By semi-quantitative RT-PCR analyses, using specific primers for *TGF-β*, it was shown that the presence of miR-21 mimic resulted in a decrease (2 fold) of *TGF-β *transcript at mRNA level, whereas miR-21 antagonist led to an increase (2.5 fold) of *TGF-β *(Figure 5) in Hela cells, respectively. Further experiments need to be conducted in order to fully verify the miR-21-*TGF-β *interaction and also the respective binding sites of miR-21 on the *TGF-β *mRNA.

## Discussion

GOmir, is a novel stand-alone application designed to elucidate the human miRNA interactions with the respective targets by using the data sets retrieved by four different computationally miRNA prediction databases, increasing in this way the validity of the results. In this study, RNAhybrid database was inserted for the first time in a computational tool that combines the results from different miRNA databases. The validity of the computational predicted targets is confirmed by recent experimental studies for certain miRNAs. For example, GOmir indicated *NFI-A as *possible target for miR-223 comparing 4 out of 4 databases (miRBase, TargetScan, RNAhybrid and PicTar-4 way). Further experimental studies by Fazi et al. confirmed this prediction and showed that miR-223 plays a crucial role during granulopoiesis by downregulating *NFI-A *[16]. GOmir provides a detailed gene description of the predicted targets accompanied by a function analysis. A reverse search initiated by a potential target can also be performed to find the predicted interacting miRNAs. Comparison with the experimentally supported target database, TarBase, is also provided. In a second next step, a detailed gene ontology clustering, including all the respective graphic charts and diagrams for the predicted targets are provided by the TAGGO module of GOmir. In this way, any group of human miRNAs and respective targets can be analysed with functional and ontology information provided, easily, in a short period of time and without using a web-based interface. The resulted common targets among different databases for a given miRNA may facilitate in selecting individuals for further experimental analyses. In this respect, our preliminary results on validating experimentally GOmir comparative predictions showed that miR-21 regulate *TGF-β *expression at mRNA level.

## Conclusion

GOmir, is a stand-alone application for studying miRNA interactions with the respective targets by using the data sets retrieved by miRBase, TargetScan, RNAhybrid, PicTar-4 way and PicTar-5 way and also the experimental one TarBase. GOmir provides a detailed gene description of the predicted targets accompanied by a function and gene ontology analyses.

## Methods

### A) JTarget

#### 1) Data acquisition

Data derived from human miRNA target predicting tools, such as TargetScan, miRanda, RNAhybrid, PicTar and TarBase were used. The TargetScan database was obtained from the TargetScan website [17]. Concerning the miRanda tool, the latest up-to-date data were downloaded from miRBase (Sanger Centre) web site [18]. Similarly, the data from RNAhybrid database were retrieved from the mirnamap website [19]. The PicTar data were obtained from the UCSC genome browser database [20]. Finally, the TarBase data were retrieved from DIANA lab website [21]. The database files were treated, in order to obtain only the human target genes. For gene description and functional analyses, three database files were downloaded from the DAVID Bioinformatics database [22], in order to implement the "Find gene description" and "Find gene function" applications and correlate in this way each gene product with a description and a function analysis, respectively.

#### 2) Data integration

The database files from the four miRNA target prediction tools were truncated to the human related information, in order to have the minimum size and all the human miRNAs were paired with the respective targets. The TargetScan database file contains miRNA families, instead of individual miRNAs. Therefore, the miRNA families file corresponding to the respective miRNAs was downloaded as well. Different gene ID systems (Refseq ID, Gene symbol, Ensembl ID) are used among different databases. In order to correlate the data among different data sets the NCBI website [23] and the DAVID Bioinformatics Database [22] were used. The downloaded files, from the DAVID database for the "Find genes description" and "Find genes functions" procedures, contained pairs of DAVID ID number/genes symbol, DAVID ID number/genes description and DAVID ID number/genes functions and were minimized to the human related information. For the functionality and performance of the application we decided to create a database with all the necessary files, which were described above. We used SQLite, a software library that implemented a self-contained, serverless, zero-configuration, transactional SQL database engine which is ideal for internal databases used for distributable, stand-alone application [24]. We imported the information from our data files in a SQLite database file, necessary for JTarget functionality which is downloaded along with the entire application installation package.

#### 3) Implementation

For JTarget, the miRNA target genes search within a single database is implemented by executing a "SELECT microRNA, target FROM database_name WHERE microRNA=microRNA_name" sql query into the database. The common targets from several database tools are found by performing inner joins among the results from the respective "SELECT" statements. JTarget comprises some more functionalities besides the miRNA common target genes prediction. After target gene selection for a given miRNA, the user can search for a description of these target genes or for their functions. These two options are implemented by executing "SELECT" queries into the entire database in order to correlate each gene with a description and/or functions, respectively. Finally, the JTarget tool is connected to the TAGGO through a button named "TAGGO", which enables the clustering of the genes. A temporary file is constructed from the output file from a miRNA target search, and then used to the TAGGO tool.

### B) TAGGO

Gene Ontology (GO) is divided into three ontology aspects which yield information common to all living organisms. Molecular Function (MF) and Cellular Component (CC) aspects answer the questions of what a gene product does and where its active form can be found, whereas the Biological Process (BP) aspect clarifies the biological objective of a gene product. Each ontology aspect is structured as a Directed Acyclic Graph (DAG), a graph with no cyclic paths (no loops) with its nodes representing the ontology terms (and their intrinsic properties) and its edges the relations between the nodes. Apparently, since the ontology is in a DAG format, each term can have more than one parents and thus have multiple paths connecting it to the root. Each GO term has a unique identifier which is used as a database cross-reference in the collaborating databases [25]. Each gene product-GO term pair is followed by an Evidence Code (EC) which indicates how an annotation to a particular term is supported. There are fourteen different ECs. In general, the higher the specificity of a term, the lower its level inside the ontology hierarchy is, and vice versa. Proteins are often annotated with terms of medium or low level in the ontology. This provides a huge amount of information that is misleading when the aim is to pin-point the main characteristics and functions of a protein or a protein set. To obtain a more global view of the attributes of a protein or a protein set, a way to assign more general terms to proteins is needed. Finding more general categories for the function and localization of a protein is equivalent of tracking the most general terms of GO which are relevant to its annotated GO terms, as more generic terms (those high in the ontology) mainly serve as abstractions which demonstrate the broader role of their children. GO is continuously expanding and improving its structure, thus serving as a dynamic ontology.

#### 1) Algorithm

TAGGO implements general terms in the GO DAG structure and automatically produces biologically meaningful results. A method to estimate the specificity of a term is the evaluation of its Information Content (IC). In Algorithmic Information Theory, the information content of an individual object is a measure of the degree of difficulty to define or describe that object [26]. In other words, high information content implies more intense effort to process an object. In biological terms, the higher the information content of a GO term, the more specific this term is and vice versa. To confine this theoretical definition into a mathematical formula, it is necessary to consider that the times a term occurs denote how general this term is. It is not even necessary to encounter the term itself but any of its children: According to the *True Path Rule*, a rule imposed in order to ensure the validity of GO entries, the pathway from a child term to its top-level parent(s) must always be true [25]. In other words, a term holds all the attributes of its ancestors and can be considered one of them. Measurement of the degree of specificity of a term is complicated by the fact that the local density of GO terms and the length of branches vary. Furthermore, "leaves" should contain the same IC, as they provide the most detailed descriptions at a given time [27].

Formalistically:

*p*(*c*), the probability of a term, is defined as the frequency of encountering this term:



where *n*_*c *_is the number of times this term (or any of its children) occur and *n*_*r *_is the number of times any term (or the root term) occurs [28]. The information content *IC*(*c*) of this term is defined as the negative logarithm of its probability:



As any leaf can occur only once, its probability is:



and its Information Content:



To normalise the Information Content of a term, its IC is divided by that of a leaf:



*IC*_*normal*_(*c*), the Normalised Information Content of a term, ranges from 0 (root) to 1 (leaf).

#### 2) Functionality

The main input file of TAGGO is a list of proteins that is experimentally produced by e.g. a large scale analysis. SwissProt accession number, gene symbol or International Protein Index (IPI) can be used to identify each protein. To load the GO structure, a GO file is used as input. OBO v.1.0, OBO v.1.2, GO (which is deprecated), OBO-XML or RDF formats are supported. To map gene products to GO terms, the organism of origin must be selected. That triggers the program to load the corresponding to the selected species GO annotation (GOA) file. Each GOA entry provides information about the database which contributes to this annotation, the date of the annotation, the object which is annotated, its synonym, its type (e.g. gene, transcript, protein), its assigned GO term, the ontology, where this term belongs to and evidence about the credibility of this annotation. Users are strongly advised to use the latest GO and GOA files which can be downloaded from the GO FTP site [29]. To increase versatility and robustness, the user has the opportunity to exclude GOA entries supported by less reliable Evidence Codes (ECs). Thus, the output file may only hold the GO annotations of the input proteins supported by well established methods. To exclude very generic terms from the classification, non-desired terms can be specified and normalised information content threshold for the three aspects can be set (default values are 4% or 0.04). Finally, the directory, where the results will be stored is chosen and all data are submitted. When the program starts running, a file which contains the GO terms of each protein for all three aspects is created. Then, the protein dataset is categorised into general GO terms, as follows: all parents of each term are found (considering all possible pathways to the root) and sorted according to ascending information content. The most general term which does not belong to the non-desired terms of the corresponding aspect is considered a category, unless all of the ten most general parents belong to the user-specified non-desired terms; in that case, the term is classified as "NO ENTRY" category. The proteins with their assigned GO categories are gathered and duplicated categories for a given protein are removed. The output is visualised in pie and bar charts which show the percentage of each GO category on the given protein dataset, in all GO aspects. Moreover, Venn lists for all aspects are created to indicate the overlaps of GO categories for the given protein dataset. These lists can be imported in VennMaster [30] to create Venn diagrams. These three types of output indicate how many proteins share a common GO category. The analysis performed describes general aspects and functions of the proteins.

### GOmir GUI implementation and prerequisites

Both tools were developed in Java Programming Language. We used the widget toolkit for Java, Swing, in order to develop the graphical user interface. As far as the JTarget database implementation is concerned, we selected the SQLite SQL database engine, which does not need any server to be installed and is very compact. In addition, Spring Framework and JFreeChart libraries were used for the implementation of TAGGO chart functionality. GOmir can be installed in any Microsoft Windows or Linux operation systems with Java Runtime Engine 1.5.0 (JRE 5.0) [31] pre installed.

### Cell line

The human Hela cell line was obtained from American Type Cell Collection (ATCC, Manassas, VA) cells were maintained in Dulbecco's modified Eagle's medium (DMEM; Sigma-Aldrich Ltd, Gillingham, Dorset, UK) supplemented with 10% (v/v) fetal bovine serum (FBS) (Gibco-BRL, Paisley, Scotland, UK).

### Transfection method

miR-21 mimic (Applied Biosystems, Foster City, CA) at a concentration of 0.4 μM, miR-21 antagonist at a concentration of 0.3 μM (Exiqon, Vedbaek, Denmark), or miR-21 scrambled antagonist at a concentration of 0.3 μM (Exiqon, Vedbaek, Denmark) were transiently transfected independently into Hela cells using the Lipofectamine 2000 reagent (Gibco-BRL) in a 1:2.5 ratio, according to the manufacturer's protocol.

### RNA exctraction and semi-quantitative RT-PCR analysis of cells

RNAs from transfected or non-transfected Hela cells were extracted with Trizol (Gibco-BRL). cDNAs were reverse transcribed from 1 mg of RNA using the MMLV reverse transcriptase enzyme and kit (Promega Ltd, Madison, WI) according to the manufacturer's instructions. PCR analysis was carried out using the following primer pairs: *hTGF-β1 *F: 5'-GCAACAATTCCTGGCGATACC-3' and *hTGF-β1 *R: 5'-GCCCTCAATTTCCCCTCCAC-3'. Semi-quantitative PCR analysis for the *hTGF-β1 *transcript was determined by using the Dolphin ID imaging software (Dolphin Imaging, Chatsworth, CA, USA) after normalizing to the *β-actin *endogenous control (primers for *β-actin *F: 5' TCTACAATGAGCTGCGTGTG 3' and *β-actin *R: 5' CAACTAAGTCATAGTCCGCC 3', respectively).

## Competing interests

The authors declare that they have no competing interests.

## Authors' contributions

M.G.R. designed and directed the research and drafted the manuscript, PZ developed JTarget and GOmir, carried out the computer simulations, participated in research design and drafted the manuscript. G.P. developed TAGGO and participated in drafting the manuscript. I.M. and K.I.P participated in discussions of the research and edited the manuscript. N.P.A directed the research and edited the manuscript. S.K. designed and directed the research and edited the manuscript. All authors read and approved the final manuscript.
